# Clinical factors associated with high PD‐L1 expression in patients with early‐stage non‐small cell lung cancer

**DOI:** 10.1111/1759-7714.15453

**Published:** 2024-09-19

**Authors:** Shuta Ohara, Kenichi Suda, Akira Hamada, Masato Chiba, Masaoki Ito, Masaki Shimoji, Toshiki Takemoto, Junichi Soh, Yasuhiro Tsutani

**Affiliations:** ^1^ Division of Thoracic Surgery, Department of Surgery Kindai University Faculty of Medicine Osaka‐Sayama Japan; ^2^ Department of Thoracic Surgery Osaka Metropolitan University Graduate School of Medicine Osaka Japan

**Keywords:** non‐small cell lung cancer, plasma fibrinogen, programmed cell death ligand 1, SUVmax

## Abstract

**Background:**

Superior outcomes have been obtained for neoadjuvant treatment with immune checkpoint inhibitors (ICI) plus chemotherapy over neoadjuvant chemotherapy alone, especially in patients with high programmed cell death ligand 1 (PD‐L1) expression. However, it is not always possible to obtain sufficient tumor specimens for biomarker testing before surgery. In this study, we explored clinical factors that can predict high PD‐L1 expression.

**Methods:**

We retrospectively enrolled 340 lung cancer patients who received pulmonary resection between 2014 and 2023 and who had PD‐L1 expression data. Chi‐squared tests and logistic regression analyses were used to identify clinical factors associated with high PD‐L1 status.

**Results:**

Univariable and multivariable analyses revealed that smoking, high maximum standardized uptake value (SUVmax) of 18F‐fluorodeoxyglucose positron emission computed tomography (18F‐FDG PET/CT), and high plasma fibrinogen are independent predictors of high PD‐L1 expression. A predictive score for high PD‐L1 expression (ranging from 0 to 3) was developed based on these parameters. Notably, only 5% of patients with a score of 0 exhibited high PD‐L1 expression, whereas this proportion increased to 53% for patients with a score of 3.

**Conclusion:**

These results showed that plasma fibrinogen, smoking history, and SUVmax are predictors of high PD‐L1 expression, providing a basis for identifying patients expected to benefit from neoadjuvant ICI treatment.

## Graphical abstract

This study identified smoking history, high SUVmax on 18F‐FDG PET/CT, and elevated plasma fibrinogen as independent preoperative predictors of high PD‐L1 expression in non‐small cell lung cancer (NSCLC) patients. A prediction score based on these three factors may help identify NSCLC patients who are likely to benefit from neoadjuvant ICI treatment, where biomarker analysis could not be performed on preoperative biopsy specimens.

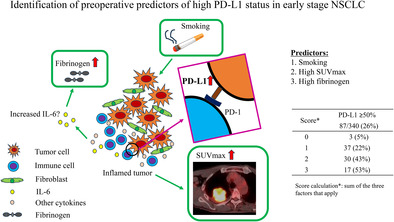

## INTRODUCTION

The clinical application of immune checkpoint inhibitors (ICIs) has revolutionized the treatment of non‐small cell lung cancer (NSCLC). After the success of ICI monotherapies or combination therapies in the advanced‐stage setting, several small‐scale, single‐arm neoadjuvant ICI studies have also reported good outcomes.^1^, ^2^, ^3^, ^4^, ^5^, ^6^, ^7^, ^8^ The Checkmate‐816 phase III study demonstrated a superior event‐free survival and pathological response in the neoadjuvant nivolumab plus chemotherapy arm compared with those of the neoadjuvant chemotherapy arm.^9^ Notably, the efficacy of nivolumab was highest in patients with high programmed cell death ligand 1 (PD‐L1) expression (50% or higher). Subsequent perioperative phase III studies (neoadjuvant ICI plus chemotherapy followed by adjuvant ICI monotherapy) demonstrated that high PD‐L1 expression is also associated with better treatment outcomes in the ICI combination arm than in the control arm,^10^, ^11^, ^12^ highlighting the importance of biomarker analyses using pre‐treatment biopsy specimens.

However, it is not always possible to obtain sufficient tumor specimens for biomarker testing before surgery. Therefore, it is necessary to identify clinical factors with predictive value for high PD‐L1 expression.^13^ For example, there is evidence that C‐reactive protein (CRP)^14^ and the maximum standardized uptake value (SUVmax) of 18F‐fluorodeoxyglucose positron emission computed tomography (18F‐FDG PET/CT)^15^, ^16^ are correlated with the PD‐L1 expression status. In this study, we hypothesized that a combination of these markers will improve the prediction accuracy in patients with early‐stage NSCLC. In addition to CRP and SUVmax, we evaluated other potential markers of inflammation, such as fibrinogen,^17^ Krebs von den Lungen‐6 (KL‐6), and the neutrophil‐to‐lymphocyte ratio (NLR).

### Patients and methods

#### Study cohort

**FIGURE 1:**
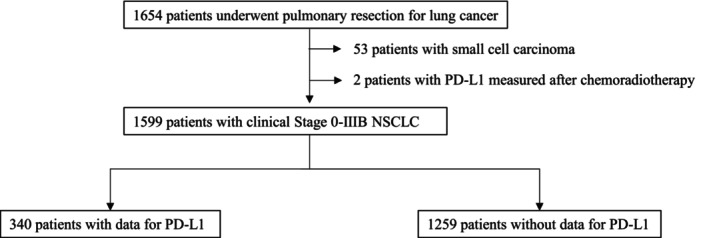
Flow diagram illustrating the study design.

Between 2014 and 2023, 1654 patients underwent curative‐intent pulmonary resection for lung cancer at our institution. After the exclusion of 53 patients with small cell carcinoma and of two patients whose PD‐L1 status has been determined after preoperative treatment, 340 patients were included in this study (Figure 1).

In our hospital, the normal values for plasma fibrinogen, KL‐6, and CRP are ≤4.0 g/L, <500 U/mL, and <0.14 mg/dL, respectively, and these were used as the cutoff values. The cutoff value for NLR was determined based on a previous report.^18^ The cutoff value for SUVmax was determined using a receiver operating characteristic (ROC) curve analysis.

The PD‐L1 status was evaluated using a 22C3 anti‐PD‐L1 antibody‐based IHC analysis (SRL, Inc., Tokyo, Japan). A tumor proportion score of ≥50% was defined as high PD‐L1 expression.

This study was approved by the Institutional Review Board at Kindai University Faculty of Medicine (31‐068). Patient consent was waived owing to the retrospective design.

#### Statistical analyses

The associations between high PD‐L1 expression and clinical characteristics were analyzed using chi‐squared tests and univariable and multivariable logistic regression analyses. ROC curve analysis was applied to determine the adequate cutoff value for SUVmax obtained by ^18^F‐FDG PET/CT. ROC curve with area under the curve (AUC) calculations were performed to determine the adequate cutoff value for the SUVmax by Youden's Index (AUC: 0.72), because the SUVmax is not an absolute number and may differ between institutions. A *P*‐value of <0.05 was considered statistically significant. All data were analyzed using JMP version 17.0 (SAS Institute, Cary, NC, USA).

## RESULTS

### Patient characteristics

The clinicopathological characteristics of the 340 patients who were included in this study are summarized in Table 1. The median age was 73 years (range 15–90 years). The majority of patients were males and smokers. We observed that 87 patients (26%) had high PD‐L1 expression (>50%), 115 patients (34%) had weak PD‐L1 expression (1–49%), and 138 patients (40%) had negative PD‐L1 expression. We also summarized patients' characteristic who were not included in this study due to the lack of PD‐L1 status (*N* = 1259). We observed that the study cohort (*N* = 340) was associated with older age, and higher SUVmax compared with patients excluded from the study.

**TABLE 1:** Clinicopathological characteristics.

Parameter	Category	Cohort included in this study (*N* = 340)	Cohort excluded from the study due to the lack of PD‐L1 status (*N* = 1259)	*p*‐value
Age	<73	149	638	0.02
	≥73	191	621	
Sex	Male	214	764	0.45
	Female	126	495	
Smoking status	Smoker	225	841	0.83
	Non‐smoker	113	411	
	Unknown	2	7	
SUVmax	<8.3	195	631	0.01
	≥8.3	129	299	
	Unknown	16	329	
CRP	<0.14 g/dL	218	779	0.45
	≥0.14 g/dL	122	480	
KL‐6	<500 U/mL	293	1111	0.35
	≥500 U/mL	45	144	
	Unknown	2	4	
NLR	<2.3	173	712	0.06
	≥2.3	167	545	
Fibrinogen	<400 mg/mL	270	993	0.95
	≥400 mg/mL	70	260	
cStage	0‐I	267	1021	0.29
	II‐IIIB	73	238	
Histology	Squamous cell carcinoma	58	291	0.056
	Adenocarcinoma	254	874	
	Others	28	94	
PD‐L1	<1%	138	–	
	1%–49%	115	–	
	≥50%	87	–	

### Univariable and multivariable analyses of the association between high PD‐L1 expression and clinicopathological factors

As shown in Table 2, univariable logistic regression analyses revealed that high PD‐L1 expression is significantly correlated with the following parameters: male sex, smoker, high SUVmax, high CRP, high KL‐6, clinical stage II‐IIIB, squamous cell carcinoma, and high fibrinogen. In a multivariable logistic regression analysis, we found that smoker (OR 2.81, 95% CI 1.16–6.79, *p* = 0.02), high SUVmax (OR 2.39, 95% CI 1.30–4.40, *p* < 0.01), and high fibrinogen (OR 2.18, 95% CI 1.06–4.48, *p* = 0.03) were independent predictors for a high PD‐L1 expression status.

**TABLE 2:** Exploration of factors associated with high PD‐L1 levels (≥50%).

Variables	OR	Univariable analysis		OR	Multivariable analysis	
		95% CI	*p*‐value		95% CI	*p*‐value
Age (≥73 vs. <73) (years)	1.14	0.70–1.87	0.59	1.4	0.76–2.40	0.31
Sex (male vs. female)	3.12	1.74–5.60	<0.01	1.4	0.63–3.16	0.4
Smoking status (smoker vs. non‐smoker)	3.69	1.94–7.02	<0.01	2.8	1.16–6.79	0.02
SUVmax (≥8.3 vs. <8.3)	3.33	1.98–5.59	<0.01	2.4	1.30–4.40	<0.01
CRP (≥0.14 vs. <0.14) (mg/dL)	1.89	1.15–3.11	0.01	1	0.54–1.99	0.91
KL‐6 (≥500 vs. <500) (U/mL)	2.21	1.15–4.25	0.02	1.5	0.73–3.20	0.27
NLR (≥2.3 vs. 2.3)	1.08	0.66–1.76	0.75	0.7	0.40–1.30	0.28
cStage (II–IIIB vs. 0–I)	2.03	1.16–3.53	0.01	1.4	0.70–2.76	0.35
Histology (squamous vs. others)	2.69	1.49–4.86	<0.01	1.4	0.72–2.82	0.31
Fibrinogen (≥400 vs. <400) (mg/dL)	2.8	1.61–4.89	<0.01	2.2	1.06–4.48	0.03

Abbreviations: CI, confidence interval; HR, hazard ratio.

### Predictive score for high PD‐L1 expression

To preoperatively predict the PD‐L1 status, we developed a risk score based on the three independent predictors that were significantly correlated with high PD‐L1 expression (i.e., smoking status, SUVmax, and fibrinogen). Patients were divided into four groups based on the score (i.e., 0 to 3); each factor was scored according to presence/absence (0/1), and the total score was obtained as the sum. Only three patients (5%) had high PD‐L1 expression in the group with score 0. The proportion of patients with high PD‐L1 expression increased as the score increased, and 53% of patients had high PD‐L1 expression in the group with score 3 (Table 3).

**TABLE 3:** Score‐based preoperative predictors of PD‐L1 expression.

Score	PD‐L1 ≥ 50%	PD‐L1 1–49%	PD‐L1 < 1%
	87/340 (26%)	115/340 (34%)	138/340 (40%)
0	3 (5%)	17 (25%)	47 (70%)
1	37 (22%)	64 (37%)	70 (41%)
2	30 (43%)	25 (36%)	15 (21%)
3	17 (53%)	9 (28%)	6 (19%)

## DISCUSSION

In the present study, we explored clinical factors that can predict high PD‐L1 expression in patients with early‐stage NSCLC. As markers, we evaluated fibrinogen, NLR, and KL‐6 in addition to the previously reported CRP.^14^ We observed that plasma CRP was significantly correlated with high PD‐L1 status, confirming the previous report.^14^ Additionally, plasma fibrinogen and KL‐6 levels were also correlated with high PD‐L1 status. However, in a multivariable analysis, fibrinogen level was the only independent predictor of high PD‐L1 expression among the plasma markers. In the multivariable analysis, we also found that smoking and SUVmax were independently correlated with high PD‐L1 expression. This result was supported by previous studies that reported a correlation between smoking status or SUVmax and PD‐L1 expression status.^15^, ^16^, ^19^ We found that more than half of patients had high PD‐L1 expression if these three parameters were positive (i.e., score 3), while only 5% had high PD‐L1 expression if these three parameters were negative (i.e., score 0).

However, it is important to note that the quantitative FDG‐PET/CT parameters, including SUVmax, differ among institutions. Therefore, the cutoff value adopted in this study, SUVmax 8.3, is not generalizable, and cutoff values should be determined at each institution. Harmonization of FDG‐PET/CT parameters^20^ can address this limitation. This study had several other limitations; in particular, it was a retrospective analysis at a single institution and PD‐L1 staining was not performed in all patients; therefore, 1259 patients were excluded from the study.

**FIGURE 2:**
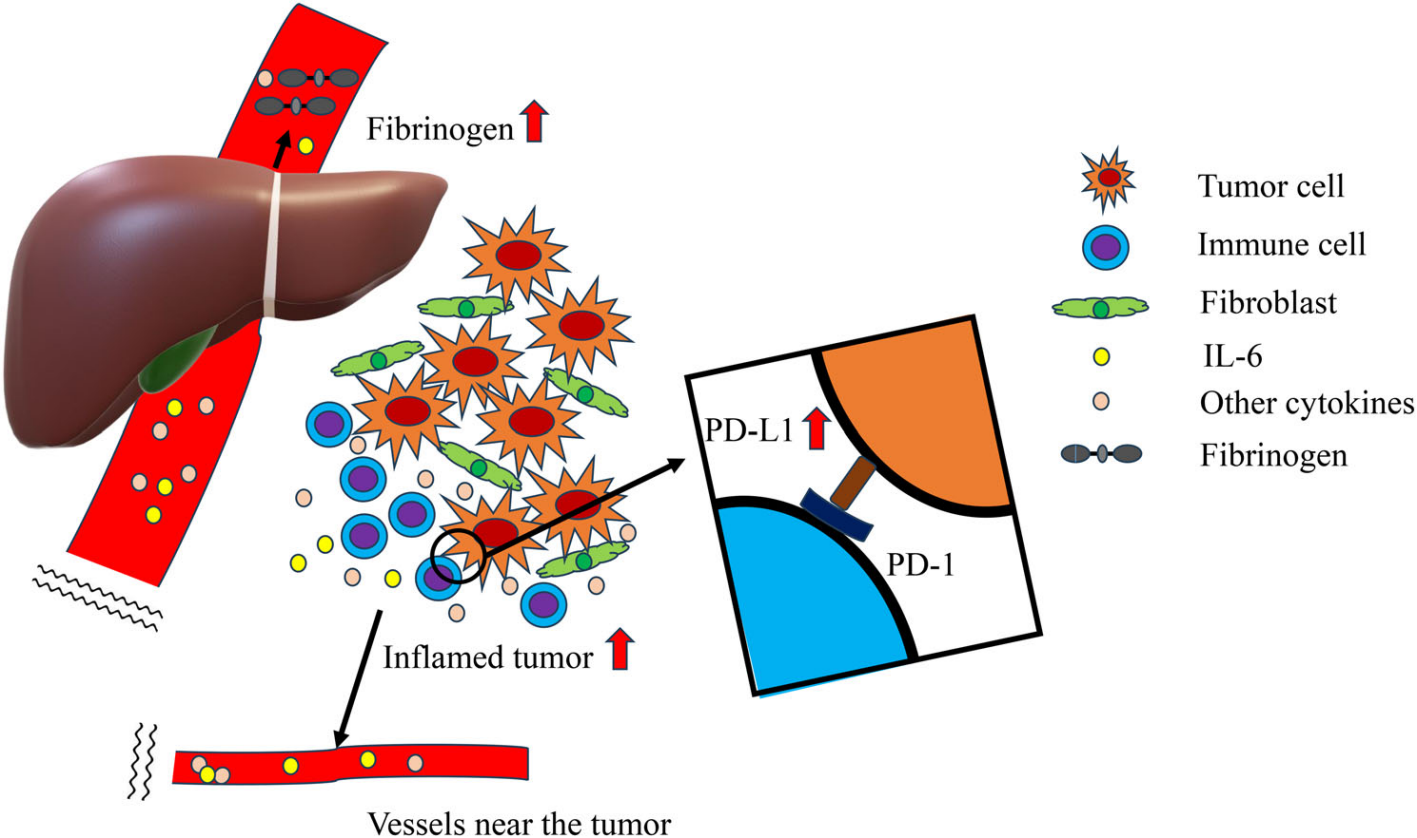
Hypothetic figure that shows a simplified potential association between plasma fibrinogen levels and the PD‐L1 expression status of the tumor.

As a basis that connect plasma fibrinogen levels and PD‐L1 expression on tumor cells, we hypothesize that interleukin‐6 (IL‐6) may have some roles on this relationship (Figure 2), although the actual circumstances between tumor immunity, tumor microenvironment, inflammation, and coagulation system would be further complex. IL‐6 is an inflammatory cytokine that plays a crucial role in regulating the immune response. Under inflammation such as inflamed tumor microenvironment with PD‐1/PD‐L1 interaction between immune cells/tumor cells, respectively, inflammatory cytokines, such as IL‐6, are released from various cells in the tumor microenvironment including fibroblasts. IL‐6 reportedly acts on several cells, including hepatocytes, to stimulate the production of CRP and fibrinogen.^21^, ^22^ Additionally, increases in inflammatory cytokines, such as IL‐6, have been shown to upregulate PD‐L1 on tumor cells.^23^

Although our study revealed three parameters that can be used to predict high PD‐L1 expression before surgery, the prediction accuracy was not 100%. In particular, despite a high specificity (94%) of the score 3 as a predictor for high PD‐L1 status, the sensitivity of this marker (20%) was not so high. Therefore, adequate biopsy to obtain high‐quality tumor samples is still desirable to determine the benefit of neoadjuvant treatment. However, our results provide a useful alternative for patients without enough tumor tissue for biomarker testing before treatment.

Despite such simple finding that can be applicable in our daily clinical practice, it should be noted that there are some limitations in this study. First, this is a retrospective analysis of single institution, and there might be a selection bias because patients characteristics who were included in this study were different in some variables from patients who were not included in this study due to the lack of PD‐L1 status (Table 1). Therefore, future validation of the findings of this study would be needed.

In conclusion, this study provides the first evidence for an association between plasma fibrinogen levels and the tumor PD‐L1 expression status (high PD‐L1 expression). We also showed that clinical factors, particularly smoking and SUVmax of FDG‐PET/CT, are effective predictors of high PD‐L1 expression, in addition to plasma fibrinogen.

## AUTHOR CONTRIBUTIONS

*Conceptualization*: Shuta Ohara and Kenichi Suda. *Methodology*: Shuta Ohara. *Validation*: Kenichi Suda. *Formal analysis*: Shuta Ohara. *Investigation*: Shuta Ohara, Kenichi Suda, Akira Hamada, Masaoki Ito, Junichi Soh, and Yasuhiro Tsutani. *Resources*: Shuta Ohara, Kenichi Suda, Masato Chiba, Masaki Shimoji, Toshiki Takemoto, and Yasuhiro Tsutani. *Writing–original draft preparation*: Shuta Ohara and Kenichi Suda. *Writing–review and editing*: All authors. *Supervision*: Kenichi Suda. *Project administration*: Kenichi Suda and Yasuhiro Tsutani. *Funding acquisition*: Shuta Ohara and Kenichi Suda.

## FUNDING INFORMATION

This study was supported by a Grant‐in‐Aid for Scientific Research from the Japan Society for the Promotion of Science (grant number 22K07291 to K.S. and 22K16583 to S.O.).

## CONFLICT OF INTEREST STATEMENT

Dr. Ohara has received honoraria from AstraZeneca. Dr. Suda has received research funding from AstraZeneca and Guardant and has received honoraria from Chugai Pharmaceuticals, Daiichi‐Sankyo, AstraZeneca, Amgen, and Taiho. Dr. Hamada has received honoraria from AstraZeneca, Chugai Pharmaceuticals, and Ono Pharmaceuticals. Dr. Tsutani has received research funding from AstraZeneca and Chugai Pharmaceuticals; has received an honoraria from AstraZeneca, Bristol Myers Squibb, Chugai Pharmaceuticals, Merck Sharp & Dohme, Ono Pharmaceuticals, and Roche; and has been on the advisory board of AstraZeneca, Chugai Pharmaceuticals, and Ono Pharmaceuticals. The remaining authors declare no conflict of interest related to the current study.

## Data Availability

The data that support the findings of this study are available from the corresponding author upon reasonable request.
